# Robust Superhydrophobic Cellulose Nanofiber Aerogel for Multifunctional Environmental Applications

**DOI:** 10.3390/polym11030495

**Published:** 2019-03-14

**Authors:** Hasan. M., Deepu A. Gopakumar, Vishnu Arumughan, Yasir Beeran Pottathara, Sisanth K. S., Daniel Pasquini, Matej Bračič, Bastien Seantier, Ange Nzihou, Sabu Thomas, Samsul Rizal, Abdul Khalil H. P. S.

**Affiliations:** 1 Chemical Education Department, Universitas Syiah Kuala, Jln. Tgk. Daud Beureueh Darussalam Banda Aceh, Banda Aceh 23311, Indonesia; muhammadhasan.kimia@unsyiah.ac.id; 2 School of Industrial Technology, Universiti Sains Malaysia, Penang 11800, Malaysia; 3 Univ. Bretagne Sud, UMR CNRS 6027, IRDL, F-56100 Lorient, France; ptyasirbeeran@gmail.com (Y.B.P); bastien.seantier@univ-ubs.fr (B.S); 4 Department of Chemistry and Chemical Engineering, Chalmers University of Technology, Kemigården 4, 412 96 Göteborg, Sweden; vishnukarumughan@gmail.com; 5 International and Inter University Centre for Nanoscience and Nanotechnology, Mahatma Gandhi University, Kottayam, Kerala 686560, India; sisanth.ks@gmail.com (S.K.S.); sabuthomas@mgu.ac.in (S.T.); 6 Chemistry Institute, Federal University of Uberlandia-UFU, Campus Santa Monica-Bloco1D-CP 593, 38400902 Uberlandia, Brazil; danielpasquini2013@gmail.com; 7 Institute of Engineering Materials and Design, University of Maribor, 2000 Maribor, Slovenia; matej.bracic@um.si; 8 Université de Toulouse, IMT Mines Albi, RAPSODEE CNRS UMR-5302, Campus Jarlard, F-81013 Albi CEDEX 09, France; ange.nzihou@mines-albi.fr; 9 Department of Mechanical Engineering, Universitas Syiah Kuala, Banda Aceh 23111, Indonesia; samsul_r@yahoo.com

**Keywords:** cellulose nanofiber aerogels, silane modification, dye removal, thermal insulators

## Abstract

The fabrication of superadsorbent for dye adsorption is a hot research area at present. However, the development of low-cost and highly efficient superadsorbents against toxic textile dyes is still a big challenge. Here, we fabricated hydrophobic cellulose nanofiber aerogels from cellulose nanofibers through an eco-friendly silanization reaction in liquid phase, which is an extremely efficient, rapid, cheap, and environmentally friendly procedure. Moreover, the demonstrated eco-friendly silanization technique is easy to commercialize at the industrial level. Most of the works that have reported on the hydrophobic cellulose nanofiber aerogels explored their use for the elimination of oil from water. The key novelty of the present work is that the demonstrated hydrophobic cellulose nanofibers aerogels could serve as superadsorbents against toxic textile dyes such as crystal violet dye from water and insulating materials for building applications. Here, we make use of the possible hydrophobic interactions between silane-modified cellulose nanofiber aerogel and crystal violet dye for the removal of the crystal violet dye from water. With a 10 mg/L of crystal violet (CV) aqueous solution, the silane-modified cellulose nanofiber aerogel showed a high adsorption capacity value of 150 mg/g of the aerogel. The reason for this adsorption value was due to the short-range hydrophobic interaction between the silane-modified cellulose nanofiber aerogel and the hydrophobic domains in crystal violet dye molecules. Additionally, the fabricated silane-modified cellulose nanofiber hydrophobic aerogels exhibited a lower thermal conductivity value of 0.037 W·m^−1^ K^−1^, which was comparable to and lower than the commercial insulators such as mineral wools (0.040 W·m^−1^ K^−1^) and polystyrene foams (0.035 W·m^−1^ K^−1^). We firmly believe that the demonstrated silane-modified cellulose nanofiber aerogel could yield an eco-friendly adsorbent that is agreeable to adsorbing toxic crystal violet dyes from water as well as active building thermal insulators.

## 1. Introduction

Due to the rapid increase in the global population, urbanization, industrialization, and agricultural activities, as well as an excessive application of chemicals, environmental pollution has drastically increased in the last decade [1]. The contamination of ground and surface water with synthetic dyes is a severe environmental hazard, and is a threat to mankind and aquatic life [2,3,4,5]. Organic and inorganic wastes have resulted in elevated volumes of polluted water, which increases the health concerns of humans and other living organisms. The release of textile dyes into water bodies can cause issues, but it is also harmful to biological organisms and ecology. Textile dyes are mainly chemical compounds that can join themselves to surfaces or objects to impart color. Over 10,000 different commercially available pigments and dyes exist, and more than 7 × 10^5^ tons per year are produced annually [6,7]. Roughly 15 wt % of dyes are left over in industrial waste water and delivered to water streams [8]. The occurrence of textile dyes in waste water leads to visual pollution, reduces water reoxygenation capacity, and leads to difficulty in waste water treatment by conventional procedures [9]. Due to the growing use of dyes, dye wastewater is creating an environmental threat, and the extraction of these pollutants from wastewater is challenging [10].

On the other hand, buildings in many developed countries have contributed more than 30% of the greenhouse gas emissions. The only cost-effective remedy for the greenhouse gas abatement is residential and commercial retrofit insulation [11]. However, the traditional insulation materials are being used in multiple layers, which has led to more complicated building materials in an adverse net to floor area. In this context, there is the need to develop high-performance, low-density thermal insulation materials. Aerogels are a class of materials that have a huge potential as high-performance thermal insulation materials for building applications due to their low thermal conductivity and acoustic insulation [12,13]. Cellulose nanofiber (CNF) aerogels as adsorbents have gained significant attention due to their enhanced adsorption capabilities, eco-friendly nature, biodegradability, and sustainability [14,15,16]. These properties could make them possible for use as an excellent material for water purification. Despite the aforementioned advantages of nanocellulose aerogels, the intrinsic hydrophilicity of the nanocellulose aerogels, which is a result of their poor integrity structure, limits their applicability in various fields, especially in water treatment. A conceivable technique to alter the hydrophilicity of CNF aerogels is to induce hydrophobicity in CNF aerogels via the modification of CNF surface with hydrophobic moieties. In recent years, most of the hydrophobization of nanocellulose aerogels was done using chlorosilanes via the chemical vapor deposition technique. Hydrophobic nanocellulose aerogels were fabricated by Hsieh et al. via the vapor deposition method using triethoxyl(octyl) silane [17]. Similarly, hydrophobic nanocellulose aerogels were produced by Sun et al. via treating hydrophilic nanocellulose aerogels into the vapor of methyltrimethoxysilane [18]. Even though the hydrophobicity of nanocellulose aerogels were enhanced by employing these chemical vapor deposition techniques, one obvious disadvantage of these methods is the inconsistent grafting distribution [14,19].

Recently, many research studies have focused on nanocellulose aerogels for the development of sustainable absorbent materials for the removal of oil from water due to its inherent properties such as high surface area, aspect ratio, and abundant hydroxyl groups [17,18,20,21,22,23,24]. Until now, only a few works have reported on the silane modification of nanocellulose. Zhang et al. reported the facile synthesis of hydrophobic nanocellulose sponges using a silylation process in water, and they demonstrated the removal of oil from water [14]. Similarly, Zhou et al. also fabricated superhydrophobic aerogels from microfibrillated cellulose using silanization reaction, and they also removed oil from water [25]. Most of the works based on silane-modified hydrophobic nanocellulose aerogels have been focused mainly on the removal of oil from water. The dye-removal studies based on CNF aerogels have been not reported extensively. Wang et al. employed crosslinked carboxylated CNF aerogels for the removal of methylene blue from the water, and the fabricated aerogel had an adsorption capacity of 127.73 mg/g against methylene blue [24]. In another work, Jian et al. employed 2, 2, 6, 6-tetramethylpiperidine-1-oxyl radical (TEMPO)-oxidized CNF aerogels for the removal of malachite green dye from water [25]. The main problem associated with the TEMPO oxidation is its toxicity and difficulty to scale up at the industry level. In this context, we aimed to prepare hydrophobic silane-modified CNF aerogels as a thermal insulation material for buildings and an effective sorbent for dye removal from water. We firmly believe that the fabricated hydrophobic silane-modified CNF aerogel will be a promising candidate for the water treatment and thermal insulation material for building applications in the near future.

## 2. Materials and Methods

### 2.1. Materials

The CNFs were extracted from eucalyptus bleached Kraft pulp and supplied in water suspension by SUZANO, Brazil. The supplied CNFs were in the range of 15 to 25 nm in diameter, with lengths of several micrometers. Methyltriethoxysilane (MTES), ethanol, and other chemicals were procured from Sigma Aldrich.

### 2.2. Fabrication of Neat CNF Aerogels

The CNF suspension (2 wt %) was frozen in an isopropyl alcohol bath at −80 °C and freeze-dried via lyophilizer (Sub-zero-SZ042, Bangalore, India) at a temperature of −50 °C and a pressure of 0.1 bar. After 36 h, CNF aerogels were obtained.

### 2.3. Fabrication of Silane-Modified CNF Aerogels

Silane-modified CNF aerogels were fabricated according to Zhou et al. [20]. The obtained neat CNF aerogels were soaked in 50 mL of ethanol in round-bottom flasks which comprised 5% silane. Then, the pH of the whole mixture was reduced to 2 with acetic acid. After this, the whole set-up was stirred at 60 °C for 90 min in an oil bath, followed by changing the pH of the mixture to 7.5 with ammonium hydroxide solution, after which it was stirred for another 1 h at 60 °C. Afterwards, the aerogels were extensively washed with methanol and ethanol to eliminate the unreacted silane from the aerogel samples. Lastly, the obtained silane modified aerogels were vacuum dried at 90 °C for 3 h.

### 2.4. Aerogel Density Assay

The regular geometrical shaped aerogel was dried for 6 h at the temperature of 60 °C, weighed, and recorded as *m*_0_. The volume “length × width × height” of the aerogel was measured by a digital caliper and recorded as *V*. Then, the digital information of the aerogel was measured three times and the average value was taken. The density (*ρ*) was calculated by:

(1) $$\rho =\frac{m_{0}}{V}$$

### 2.5. Scanning Electron Microscopy

The morphology of fabricated pristine CNF aerogel and silane-modified aerogel were investigated via SEM (Carl Zeiss, EVO MA10, Feldbach, Switzerland ). Gold sputtering was done on the both samples prior to testing them in an argon atmosphere. SEM (Hitachi, SU8010, Tokyo, Japan) coupled with energy-dispersive X-ray spectroscopy (EDS) was employed to investigate the morphology of both fabricated aerogels.

### 2.6. X-Ray Diffraction Analysis

X-ray diffraction analysis were done on the samples to check whether the silane-modified CNF aerogel maintained their cellulose I structure. In order to investigate this, Shimadzu XRD-6000 was employed to obtain the XRD patterns of pristine CNF aerogel and silane-modified CNF aerogel at a scan rate of 2°/min from 2θ = 5° to 40°.

### 2.7. Fourier Transform Infrared Spectroscopy (FTIR)

In order to obtain the FTIR spectra of neat CNF aerogel and silane-modified CNF aerogel, a Fourier transform infrared spectrophotometer (Shimadzu) was used from 400 to 4000 cm^−1^. The ATR method was used to obtain the spectras.

### 2.8. Static Contact Angle and Surface Free Energy

Static contact angle (SCA) measurements of neat CNF aerogel and silane-modified CNF aerogel were performed using a goniometer system OCA15+ (Dataphysics, Filderstadt, Germany). Four different liquids such as Milli-Q water, diiodomethane, formamide, and ethylene glycol were employed to take the measurements at room temperature via a drop volume of 3 L. Each SCA value is an average of five drops per surface. The acid base approach of van Oss and Good was used to calculate the total SFE (·) of both fabricated aerogels. Further details on the static contact angle and surface free energies of the liquids that had used for the measurements are given in the Table S1 in Supplementary Information.

### 2.9. UV-Visible Spectrophotometer

In order to quantify the solute concentrations in the feed and final solutions, the UV-vis technique was employed. The removal of dye was studied using (Femto Mod. 800X I) through absorbance. Initial and final solutions were transferred into a PMMA disposable cuvette, and absorbance was measured at 590 nm. The spectrum of a cuvette with distilled water was used as the reference in order to standardize the absorbance of samples.

### 2.10. Evaluation of Adsorption Efficiency of Silane-Modified CNF Aerogels against Positively Charged Dyes

Here, we employed crystal violet dye as a toxic textile dye to determine the adsorption capacity of silane-modified CNF aerogel. The estimation of the adsorption efficiency of the silane-modified CNF aerogel was done by using the method of Gopakumar et al. [9]. Firstly, 0.05 g of the silane-modified CNF aerogel was dipped in 30 mL of CV (10 mg/L, pH 7.0) solution on a shaking bed for 10 to 250 min at room temperature. The adsorption capacity of silane-modified CNF aerogel against CV dye was determined using UV-visible spectrophotometer via optical absorption at 590 nm from the concentration change of the CV solution before and after adsorption.

### 2.11. Thermal Conductivity Measurements

**Figure 1:**
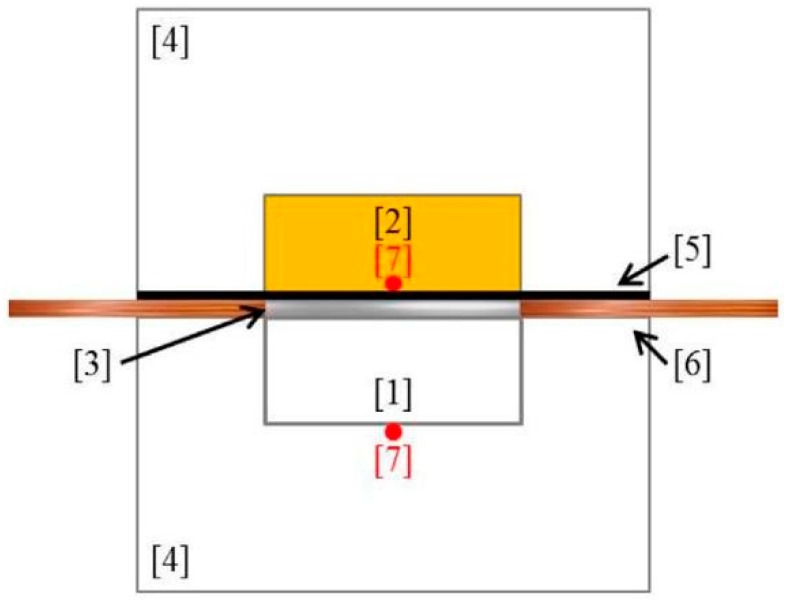
Homemade hot-strip technique. The sample [1] is facing a reference sample made of PU [2] in a temperature-controlled aluminum device [4]. An insulator [5] is protecting the reference sample from the heat flow delivered by the hot-strip technique [3] via electrodes [6]. Two thermocouples [7] allow measuring the temperature of both sides of the sample.

Thermal conductivity has been characterized by a home-made hot-strip technique described elsewhere (Figure 1) [26,27]. The sample is placed in a temperature-controlled aluminum cavity. The sample is facing a symmetrical reference cavity filled with polyurethane foam. A hot-strip in contact with the sample delivers a heat flow via electrodes. Then, the temperature is measured on both sides of the sample with two thermocouples. This device allows measuring the conductance of the sample via Equation (2):

K = Q/ΔT = U × I/(T_2_ − T_1_) = U × V/(R (T_2_ − T_1_)) (2)

where U is the tension through the strip, V/R is the intensity in the strip, and T1 and T2 are the temperatures at both sides of the sample. U and V are obtained with a multimeter, and R is equal to 50 Ω.

Then, the thermal conductivity, λ, is calculated from the measured conductance, K, via Equation (3):

λ = A·K + B(3)

where A and B are constants determined by a calibration curve. The calibration curve is done with materials (wood, cork, polycarbonate and polyurethane samples) for which the conductance and the thermal conductivity have been determined with other techniques.

### 2.12. Evaluation of the Mechanical Properties

Compression tests of both neat CNF aerogel and silane-modified CNF aerogel were done using the universal testing machine Tinus Olsen 50 KT coupled with an 100-N load cell. Both fabricated aerogels were cut into a rectangular piece (12 mm × 10 mm × 18 mm). The samples were compressed with a speed of 1 mm/min up to 40% compressive stress. The measurements were taken five times.

## 3. Results and Discussion

### 3.1. Morphology of CNF Aerogel and Silane-Modified CNF Aerogel

**Figure 2:**
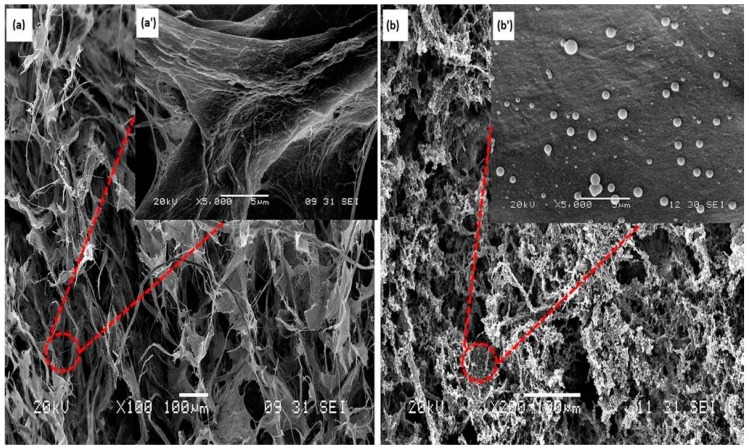
SEM images of the (**a**) neat cellulose nanofiber (CNF) aerogel; (**a’**) magnified image of neat CNF aerogel; (**b**) silane-modified CNF aerogel; and (**b’**) magnified image of silane-modified CNF aerogel.

The morphologies of the neat CNF aerogels and silane-modified CNF aerogels were investigated via SEM. From the Figure 2a,a’, it can be clearly seen that the neat CNF aerogels were porous in nature. In the magnified image (Figure 2a’), the porous CNF aerogel was self-aggregated into porous and sheet-like structures. This structure has already been seen elsewhere, and was coming from the freeze-drying formation process of aerogel. It has already been described in previous work [28]. Briefly, when freezing, the CNF of the starting suspension was aggregated at the border of the growing ice crystals. During this process, the aggregated CNF interacted via hydrogen bounds and/or entanglement. This was forming “sheets” of CNF that were maintained after removing the solvent. The highly similar microstructures in neat CNF aerogels and silane-modified CNF aerogels are shown in Figure 2, and indicate that the silane treatment could not affect the porous structures of the neat CNF aerogels. From the Figure 2b,b’, it can be clearly seen that some polysiloxane particles were formed on the surface of the CNF aerogels after the silane modification, which resulted in the hydrophobicity in the fabricated silane-modified CNF aerogel.

**Figure 3:**
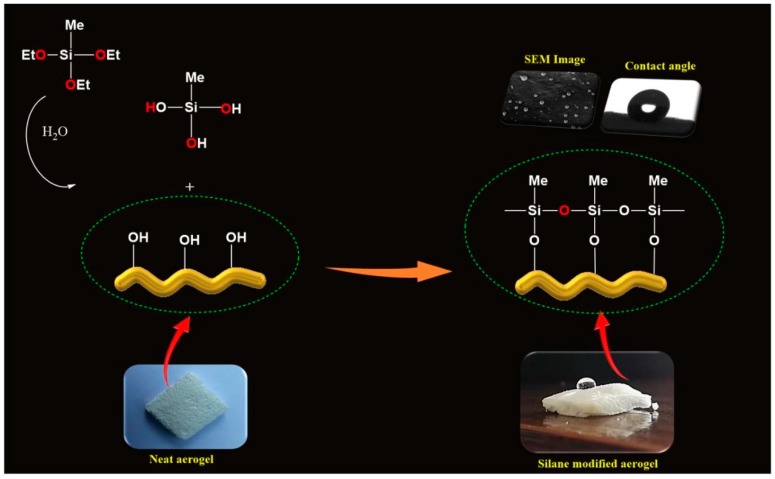
The strategy for the formation of polysiloxane particles during the silane modification of CNF aerogels.

The protocol for the formation of polysiloxane particles is illustrated in Figure 3. Here, the silanols might react with the surface hydroxyl groups of CNF aerogels, which resulted in the hydrolysis of MTES in the aerogel. A covalently attached silane coating was formed on the surface of the CNF aerogels due to the reaction between hydroxyl groups in the CNF aerogel and silanols and the self-polymerization of silanols.

### 3.2. FTIR Studies

**Figure 4:**
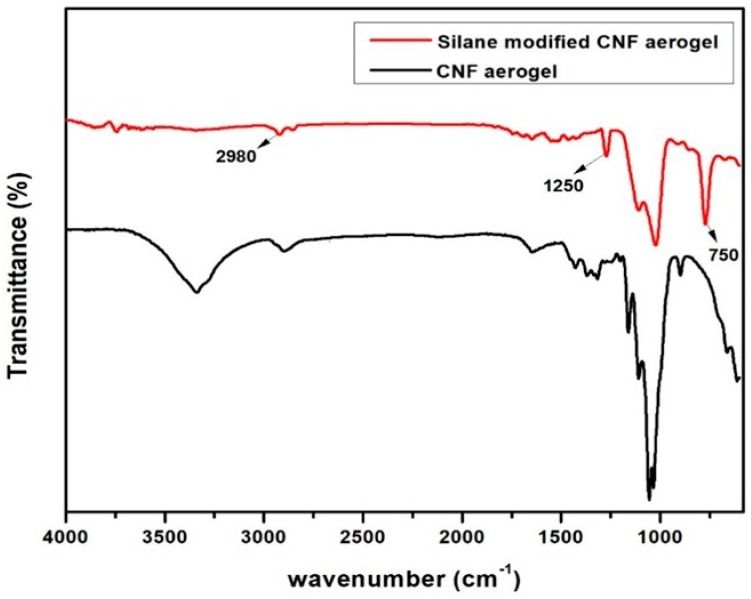
Fourier transform infrared (FTIR) spectra of neat CNF aerogel and silane-modified CNF aerogel.

In order to confirm the modification of the aerogels by polysiloxane, an FTIR experiment was carried out. Figure 4 illustrates the FTIR spectra of neat CNF aerogels and the silane-modified CNF aerogel.

Figure 4 shows that a strong band arises at 750 cm^−1^ and 2980 cm^−1^, which was attributed to the vibration characteristic of the CH3 in silane [20]. Moreover, the vibration characteristic at 1250 cm^−1^ was due to the Si−CH3 bending vibration. The characteristic peaks between 1000–1130 cm^−1^ was attributed to the overlapping between the C–O bonds of cellulose with Si–O–Si bonds in the siloxane [20]. All of these results suggested that the silane modification was successfully done on CNF aerogels.

### 3.3. X-Ray Diffraction Studies

**Figure 5:**
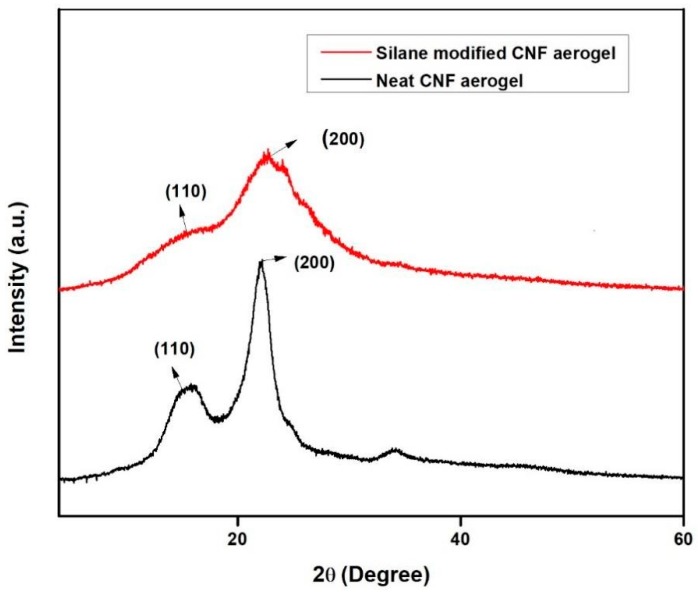
The XRD patterns of the neat CNF aerogel and silane-modified CNF aerogel.

The XRD spectra of the neat CNF aerogel and silane-modified CNF aerogel are shown in Figure 5. The diffraction peaks of neat CNF aerogel show 2Ɵ = 15.3° and 22.6°, with respect to the (110) and (200) planes, which are characteristically attributed to the cellulose type I structure [29].

Similarly, the XRD spectra of silane-modified CNF aerogel also exhibited the peaks at 2Ɵ = 15.3° and 22.6°. From the XRD pattern of silane-modified CNF aerogel, it could be concluded that the silane modification only affected the hydroxyl groups on the surface of the CNF aerogels. The XRD patterns of the silane-modified CNF aerogel that are displayed are characteristic of the cellulose I structure, demonstrating that the cellulose I structure was conserved even after the silane modification of CNF aerogels. However, there was a decrement in the intensity of the XRD patterns of the silane-modified CNF aerogel when compared to the neat CNF aerogel, which clearly indicates the successful coating of polysiloxane on the surface of the neat CNF aerogels. The Segal method was used to determine the crystallinity index (CrI) of the silane-modified CNF aerogel and neat CNF aerogel, as given in Equation (4) [30].

CrI = [(I_200_ − I_AM_)/I_200_] × 100 (4)

where CrI denotes the crystallinity index, I200 is the maximum intensity of diffraction at 2Ɵ = 22.18°, and IAM is the intensity of diffraction at 2Ɵ = 15.36°. The obtained crystallinity index of the silane-modified CNF aerogel and neat CNF aerogel samples were 34.4% and 69.45%, respectively.

### 3.4. Interface Interaction between CNF and MTES

**Figure 6:**
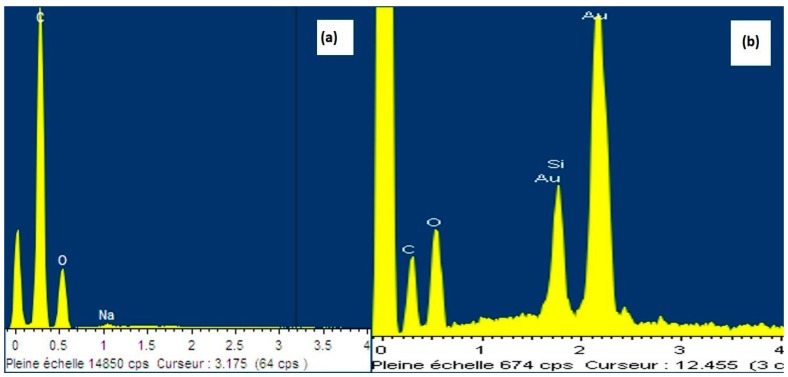
Energy-dispersive X-ray (EDAX) spectra of the neat CNF aerogel and the silane-modified CNF aerogels. The gold picks are due to the gold coating needed for SEM images.

Energy-dispersive X-ray (EDAX) spectra was also employed to study the silanization on the surface of the neat CNF aerogels. Figure 6 shows the EDAX spectrum of surface of the neat CNF aerogel and silane-modified CNF aerogels. Both neat and silane-modified aerogels displayed the carbon and oxygen peaks. This was due to the cellulose chain in CNF aerogels. In the case of silane-modified aerogels, the % mass of silane was 5.98 (as given in the Table 1), which was attributed to the successful coating of polysiloxane particles onto the surface of the CNF aerogel. From Table 1, it can be confirmed that silane modification was successfully done on the surface of the CNF aerogels.

### 3.5. Dye Adsorption Capacity

**Figure 7:**
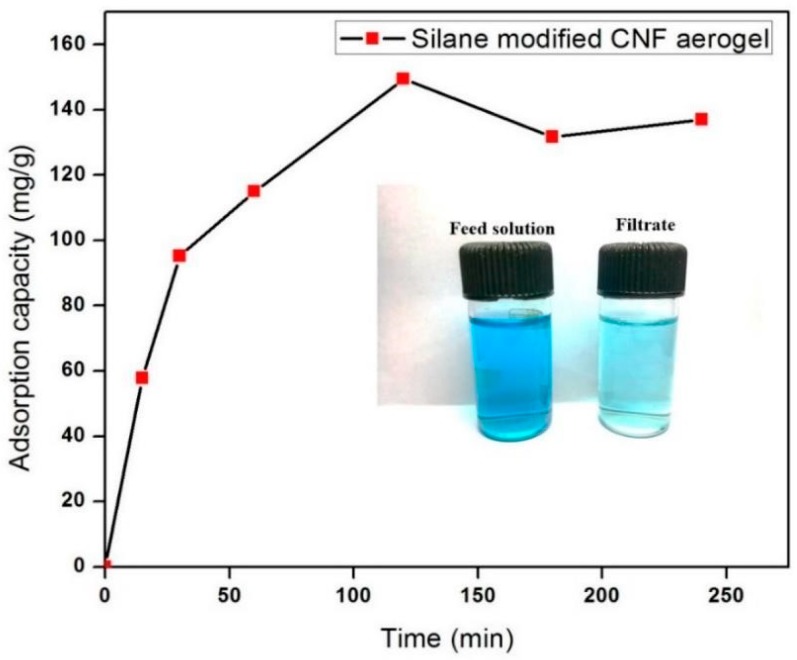
Adsorption capacity of silane-modified CNF aerogel against crystal violet dyes.

To investigate the adsorption efficiency of the fabricated silane-modified CNF aerogel, we used crystal violet (CV) dye as a toxic textile dye. Figure 7 shows the adsorption efficiency of the fabricated silane-modified CNF aerogel against crystal violet dye (10 mg/L CV) as a function of time. It was found that the CV adsorption of the silane-modified CNF aerogel was 150 mg/g of the aerogel from 10 mg/L of CV solution, which was higher than those of Ma et al. and Gopakumar et al., who reported that the adsorption of crystal violet dye were 3.8 mg/g and 3.984 mg/g using a poly(acrylonitrile) (PAN) scaffold nanofibrous membrane with cellulose nanowhiskers and Meldrum’s acid CNF-based PVDF membrane, respectively [9,31].

**Figure 8:**
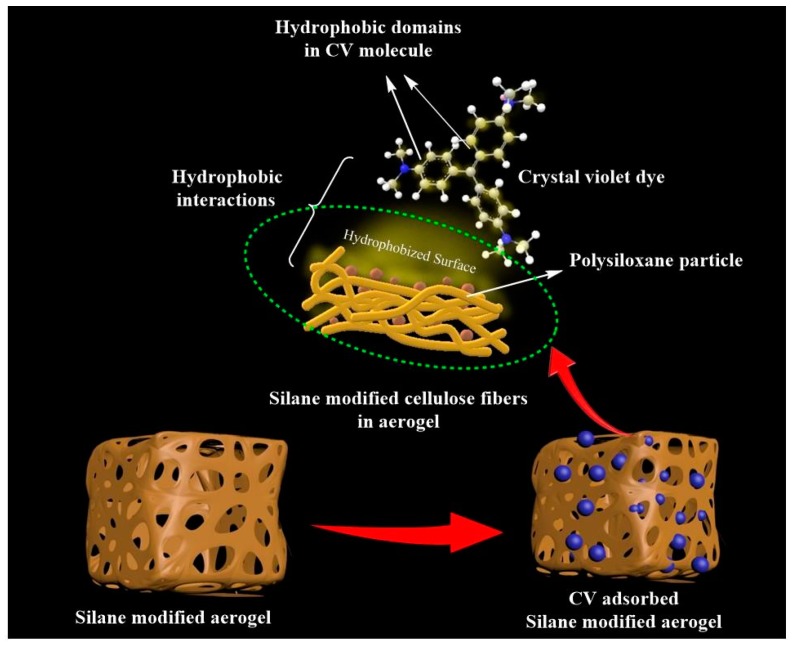
Proposed mechanism of crystal violet removal by silane-modified CNF aerogel.

This observed high sorption efficiency value of demonstrated hydrophobic cellulose aerogels toward CV can be attributed to the possible short-range hydrophobic interaction between silane-modified cellulose aerogel and hydrophobic domains in CV molecules [32,33] (mechanism shown in Figure 8). The adsorption of CV onto the silane-modified CNF aerogel had attained equilibrium after 2 h. In this context, it can be concluded that the fabricated highly hydrophobic silane-modified CNF aerogel showed substantial adsorption capacity against toxic crystal violet dyes.

### 3.6. Contact Angle Studies

**Figure 9:**
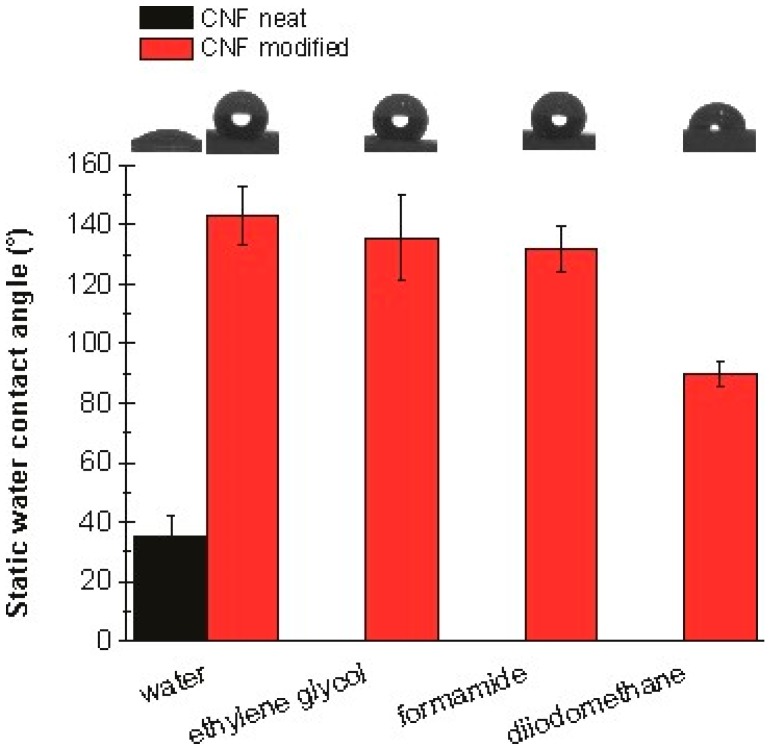
Static contact angles of water, ethylene glycol, formamide, and diiodomethane on neat CNF aerogel and silane-modified CNF aerogel.

The static contact angle measurements reveal changes in the wettability and surface free energy of silane-modified CNF aerogels in comparison to neat CNF aerogels. As can be seen in Figure 9, the water static contact angle (SCA) of the neat CNF aerogel drastically increases when modified with silane. The neat CNF aerogel exhibited a water SCA of 35°, making it hydrophilic, while the silane-modified aerogel exhibited a value of over 130°, making it superhydrophobic. The SCA of the silane-modified CNF aerogel was also measured for ethylene glycol, formamide, and diiodomethane, as shown in the Figure 9. The lowest SCA value was observed for diiodomethane (below 90°). Diiodomethane exhibited the highest value of all the chosen liquids, which is an indicator of the dispersive interactions between this liquid and the thin film arising from the hydrophobic nature of the aerogel, confirming the high values of the water SCA.

Measuring the SCA of four different liquids also allowed us to determine the surface free energy (SFE) of the silane-modified CNF aerogel. The SFE values for the chosen liquids can be found in the supplementary information (SI1). The surface free energy of the silane-modified CNF was below 13 mJ m^−2^, which is an indicator of its low surface reactivity. It also reflects the superhydrophobic nature of the thin film. It was also notable that the γ $\gamma_{s}^{LW}$ part (12.54 ± 1.99 mJ m^−2^) of the $\gamma_{1}^{TOT}$, which is responsible for the dispersive interactions and the hydrophobic nature of the aerogel, contributes to 100% of the $\gamma_{1}^{TOT}$, while the $\gamma_{s}^{+}$ and $\gamma_{s}^{-}$ parts (0.00 ± 0.00 mJ m^−2^), which are responsible for the polar interactions, don’t contribute or contribute insignificantly to the $\gamma_{1}^{TOT}$. 

### 3.7. Mechanical Strength of Fabricated Silane-Modified CNF Aerogel

**Figure 10:**
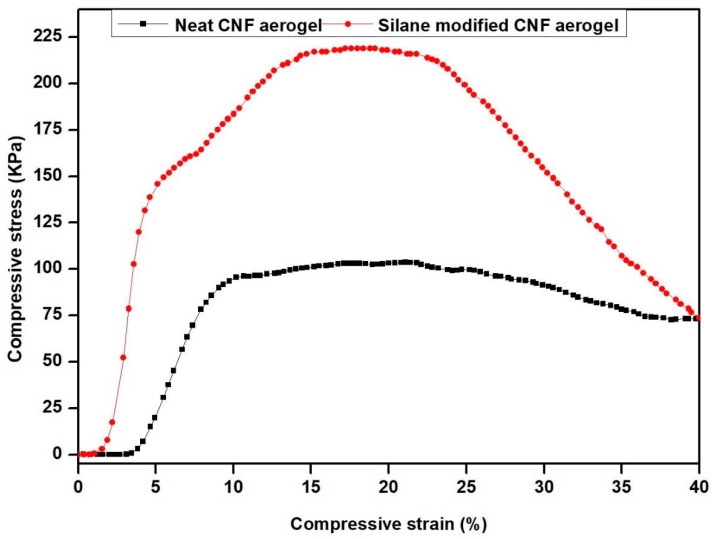
Compressive stress vs. strain curves of silane-modified CNF aerogel and neat CNF aerogel.

The mechanical properties of the aerogels are very relevant for water purification and thermal insulating applications. The mechanical properties of neat CNF aerogel and silane-modified CNF aerogel were evaluated by compression tests (Figure S1 of supporting information: Experimental set-up used for compression tests). Figure 10 shows the compressive stress versus strain curves of silane-modified CNF aerogel and neat aerogel. From Figure 10, it was shown that at 20% strain, the silane-modified CNF aerogel showed an enhanced compressive stress of 210.12 KPa, whereas the neat CNF aerogel showed 102.10 KPa. The enhanced compressive stress value of the silane-modified CNF aerogel compared with neat CNF aerogel was due to the presence of cross-linked Si–O–Si bonds of polysiloxane in the silane-modified CNF aerogels.

### 3.8. Thermal Conductivity Values of Neat CNF Aerogel and Silane-Modified CNF Aerogel

In order to explain the thermal conductivity of neat CNF aerogel and silane-modified CNF aerogel, it is necessary to describe how to evaluate the thermal conductivity.

It is usually admitted that the thermal conductivity is the result of three independent contributions: one conductive contribution due to the heat transfer in the material solid skeleton, which is called solid conduction; one conductive contribution due to the heat transfer due to gas molecule collision, which is called gas conduction; and one radiative contribution due to the heat transfer induced by radiation interactions with the material, which is called radiative conduction. The higher the density, the easier the heat transfer through solid skeleton, the higher the solid contribution, and the higher the thermal conductivity. In the present case, the density of the neat CNF aerogel and silane-modified CNF aerogel were 0.041 g/cm^3^ and 0.054 g/cm^3^, respectively. The obtained thermal conductivity of the neat CNF aerogel (0.034 W·m^−1^·K^−1^) was slightly lower than that of the silane-modified CNF aerogel (0.037 ± 0.001), which was due to the difference in the densities. It was very interesting to note that the obtained thermal conductivities of the neat CNF aerogel and silane-modified CNF aerogels were comparable and lower than that of commercial insulators such as mineral wools (0.040 W·m^−1^·K^−1^) and polystyrene foams (0.035 W·m^−1^·K^−1^) [34]. Table 2 shows some of the reported thermal conductivity values of cellulose-based materials. From Table 2, it is evident that the obtained thermal conductivity value of the silane-modified CNF aerogel was comparable to and lower than that found in other reports. From the results, it can be concluded that the silane-modified CNF aerogel could be a potential candidate as a thermal insulator for building applications due to its lower thermal conductivity and superior mechanical properties.

## 4. Conclusions

We developed a silane-modified hydrophobic CNF aerogel for the adsorption of crystal violet dyes from water and active building thermal insulators. The hydrophobicity was induced by the successful formation of polysiloxane particles on the surface of the CNF aerogels via silane modification, which resulted in superhydrophobic CNF aerogels with a water contact angle greater than 130°. The demonstrated simple silanization technique to modify the surface of CNF aerogels in liquid phase is eco-friendly, cost effective, rapid, and commercializable at the industrial scale. Moreover, the fabricated silane-modified CNF aerogel could effectively eliminate the crystal violet dyes from water. It was demonstrated that with 10 mg/L of crystal violet solution, the fabricated modified aerogel showed a high adsorption value of 150 mg/g of the aerogel. This high adsorption value of the silane-modified cellulose aerogel toward crystal violet dye was due to the short-range hydrophobic interactions between the silane-modified hydrophobic cellulose aerogels and hydrophobic domains in the crystal violet dye molecules. Furthermore, the fabricated silane-modified CNF hydrophobic aerogels exhibited a lower thermal conductivity value of 0.037 W·m^−1^·K^−1^. So, it is auspicious to use as an active building insulator. Furthermore, this demonstrated work provides insight into a new platform for the applicability of hydrophobic CNF aerogels for the removal of the wide range of toxic textile dyes from water.

## Figures and Tables

**Table 1:** Elemental composition of the silane-modified CNF aerogels.

Element	Silane-Modified CNF Aerogel % Mass	Neat CNF Aerogel % Mass
C	42.00	73.16
O	52.02	26.84
Si	5.98	0.00
Total	100.00	100.00

**Table 2:** Thermal conductivity values of some cellulose-based materials.

Materials	Thermal Conductivity (W·m^−1^·K^−1^)	References
Ag2O/Nanofibrillated cellulose aerogels	0.072	[35]
Silica/cellulose hybrid aerogels	0.040	[36]
Nanofibrillated cellulose/graphene films	0.042	[37]
Multi-scale cellulose bio-aerogel composites	0.023	[38]
CNF/AlOOH aerogel	0.039	[39]
Silane-modified CNF aerogel	0.037	This work

## Supplementary Materials

The following are available online at https://www.mdpi.com/2073-4360/11/3/495/s1, Figure S1: Experimental set-up for compression tests. Table S1: $\gamma_{l}^{TOT}$, $\gamma_{l}^{LW}$, $\gamma_{l}^{+}$, $\gamma_{l}^{-}$ values for water, ethylene glycol, formamide, and diiodomethane as chosen in this work.
