# Supplementary material for: Robust Superhydrophobic Cellulose Nanofiber Aerogel for Multifunctional Environmental Applications
Source: Polymers (Basel). 2019 Mar 14;11(3):495. doi: 10.3390/polym11030495 (PMC6473771; doi:10.3390/polym11030495)
Supplement: Supplementary file 1 [file polymers-11-00495-s001.pdf]

Supporting Information

(SI1)

The van Oss and Good approach to determine the SFE splits the total SFE (γ_s^{TOT}) in the dispersive Lifshitz–van der Waals interactions (γ_s^{LW}) and the polar Lewis acid–base interactions (γ_s^{AB}) as shown in Equation SI1.1. The acid base interactions are further divided into electron donor (γ_s^-) and electron acceptor (γ_s^+) parts (Equation SI1.2):[1]

$$\gamma_s^{TOT} = \gamma_s^{LW} + \gamma_s^{AB}, \text{ (SI1.1)}$$

$$\gamma_s^{AB} = 2 \cdot \left(\sqrt{\gamma_s^+ \gamma_s^-}\right). \text{ (SI1.2)}$$

The γ_s^+ characterises the tendency of a surface to interact with liquids that are electron donors, while the γ_s^- characterises its tendency to interact with liquids that are electron acceptors.[2] Further, the work of adhesion W_{ad} can be related to the measured contact angles (Θ) by taking Young's equation into account:[3]

$$W_{ad} = (1 + \cos\Theta)\gamma_l^{TOT} = 2\left(\sqrt{\gamma_s^{LW}\gamma_l^{LW}} + \sqrt{\gamma_s^+\gamma_l^-} + \sqrt{\gamma_s^-\gamma_l^+}\right), \text{ (SI1.3)}$$

Where γ_l^{TOT} is the total surface tension of the liquid, γ_s^{LW} and γ_l^{LW} are the disperzive Lifshitz–van der Waals parts of the liquid and the solid, respectively, and $\gamma_s^+\gamma_l^-$ and $\gamma_s^-\gamma_l^+$ are the acid-base contributions of either the solid or the liquid phase. To resolve the equation one must use at least three test liquids with known γ_l^{TOT} , γ_l^{LW} , γ_l^- , and γ_l^+.[3] Young's equation can be applied for ideal surfaces, which are flat, inert, and chemically homogeneous, but doesn't estimate the energy of a surface which is not ideal. The SFE calculations in this work were carried out for an ideal surface.

Table S1.1: γ_l^{TOT}, γ_l^{LW}, γ_l^+, γ_l^- values for water, ethylene glycol, formamide, and diiodomethane as chosen in this work.

**Liquid**	γ_l^{TOT}	γ_l^{LW}	γ_l^+	γ_l^-	Author
Water	72.8	21.8	25.5	25.5	Erbil
Ethylene glycol	48.0	29.0	3.0	30.1	Van OSs
Formamide	58.0	39.0	2.28	39.6	Van Oss
Diiodomethane	50.8	50.8	0.7	0.0	Gonzáles-Martin

Compression experiments

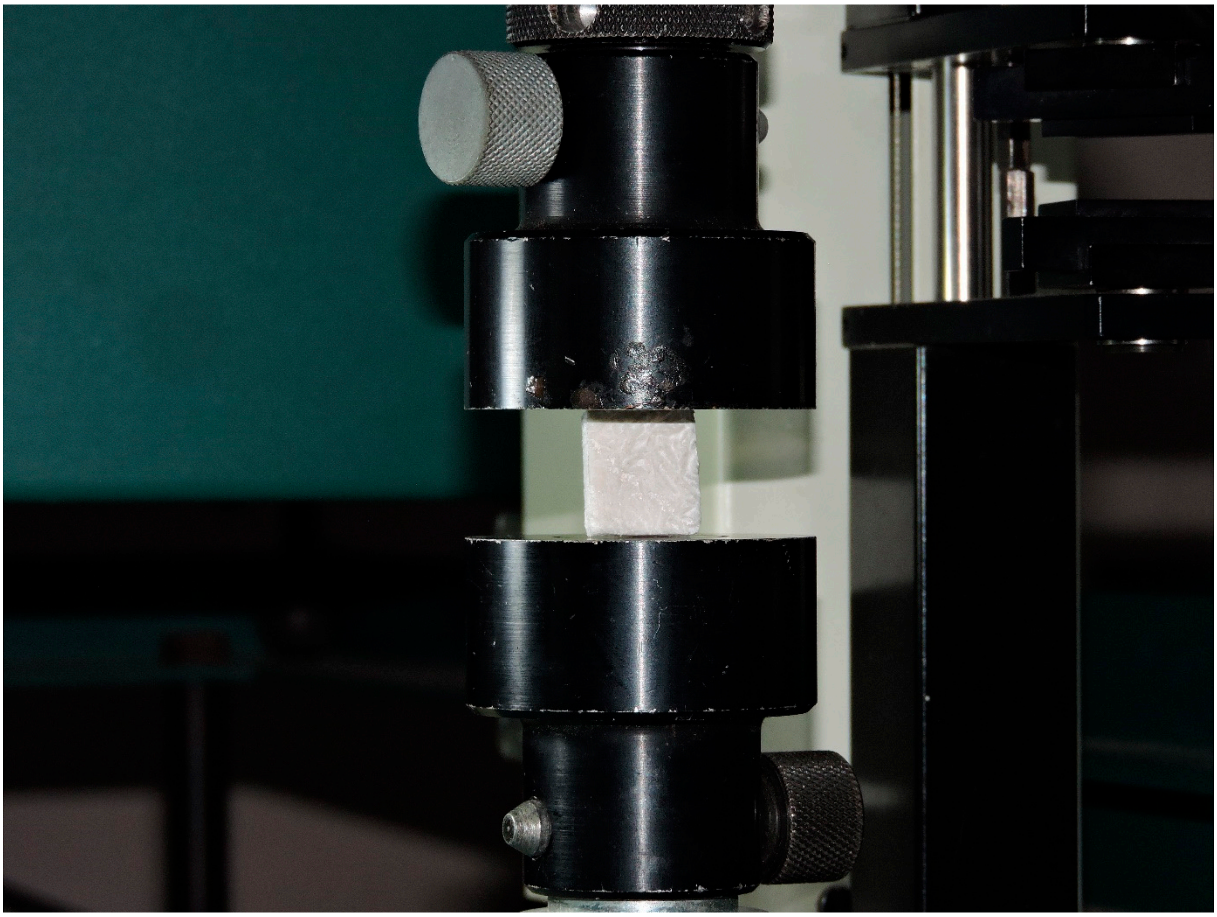

Figure S1: Experimental set-up for compression tests.

References

1. Mohan, T. *et al.* Wettability and surface composition of partly and fully regenerated cellulose thin films from trimethylsilyl cellulose. *J. Colloid Interface Sci.* **358,** 604–610 (2011).
2. Peršin, Z., Stenius, P. & Stana-Kleinschek, K. Estimation of the surface energy of chemically and oxygen plasma-treated regenerated cellulosic fabrics using various calculation models. *Text. Res. J.* **81,** 1673–1685 (2011).
3. Mohan, T. *et al.* Cationically rendered biopolymer surfaces for high protein affinity support matrices. *Chem. Commun.* **49,** 11530 (2013).
